# An Evaluation of the Impact of Increasing the Awareness of the WHO Access, Watch, and Reserve (AWaRe) Antibiotics Classification on Knowledge, Attitudes, and Hospital Antibiotic Prescribing Practices

**DOI:** 10.3390/antibiotics12060951

**Published:** 2023-05-23

**Authors:** Salam Abu-Ajaleh, Feras Darwish Elhajji, Shatha Al-Bsoul, Rana Abu Farha, Fawzi Al-Hammouri, Amer Amer, Ahmed Al Rusasi, Sayer Al-Azzam, Mohammad Araydah, Mamoon A. Aldeyab

**Affiliations:** 1 Faculty of Pharmacy, Applied Science Private University, 166, Amman 11193, Jordan; salamfateh97@gmail.com (S.A.-A.); f_elhajji@asu.edu.jo (F.D.E.); r_abufarha@asu.edu.jo (R.A.F.); 2 The Specialty Hospital, 930186, Amman 11193, Jordan; shatha.b@specialty-hospital.com (S.A.-B.); info@specialty-hospital.com (F.A.-H.); pharm.amer1982@gmail.com (A.A.); 3 Jordan Pharmacists Association, 1124, Amman 11118, Jordan; alrusasi@gmail.com; 4 Clinical Pharmacy Department, Faculty of Pharmacy, Jordan University of Science and Technology, Irbid 22110, Jordan; salazzam@just.edu.jo; 5 Princess Basma Teaching Hospital, Irbid 22110, Jordan; mohaari98@gmail.com; 6 Department of Pharmacy, School of Applied Sciences, University of Huddersfield, Huddersfield HD1 3DH, UK

**Keywords:** AWaRe classification, access, watch, reserve, WHO, antibiotics resistance, antimicrobial stewardship, physicians, pharmacists, clinical practice

## Abstract

The study aims to determine the effect of enhancing knowledge and awareness of the WHO Access, Watch, and Reserve (AWaRe) antibiotics classification on hospital clinical staff’s knowledge, attitudes and antibiotic prescribing practices. A pre-post-intervention study design was employed. The intervention was an educational activity that involved teaching physicians and pharmacists about the AWaRe classification and the risk of antibiotic resistance. A questionnaire was administered to clinical staff pre-and post-intervention. In the pre-interventional stage, 78.5% of participants stated they had not heard about the AWaRe classification of antibiotics. After receiving the intervention: the knowledge regarding the meaning and purpose of AWaRe classification of antibiotics increased from 39.1% to 75.4%; the percentage of participants who agreed with following the AWaRe classification of antibiotics in their practice increased from 21.7% to 58.5%; and the percentage of participants who agreed that AWaRe classification of antibiotics can suggest safe choices of antibiotics increased from 56.5% to 90.8%. Hospital antibiotic use of the Access group increased by 6.6% from pre- to post-intervention. The use of the Watch group and Reserve group decreased post-intervention by 1.7%, and 43.1%, respectively. This study showed important gaps in knowledge and attitudes towards AWaRe, highlighting the need for increasing the awareness of the AWaRe tool amongst healthcare practitioners to ensure rational use of antibiotics.

## 1. Introduction

Antimicrobial agents are essential and life-saving drugs, and the discovery, development, and use of antibiotics in the medical field have been the most remarkable medical breakthroughs over the years [1,2]. Antimicrobial resistance (AMR) is considered a critical challenge to the world’s health system because they decrease the efficacy of drugs and increase morbidity and mortality in critical cases [3]. The acceleration of resistance due to widespread use, overuse, misuse, and the shift toward broad-spectrum antimicrobials has raised a critical concern for the world’s public health [4,5]. Available evidence proves a strong relationship between levels of antimicrobial consumption and AMR [6,7]. The unnecessary consumption and inappropriate use of antimicrobial agents, not considering the spectrum of activity upon prescribing, and the improper use of dosage form, route of administration, or duration of an antimicrobial can increase resistance [8]. The attention of health organizations and governmental authorities worldwide has gradually increased to this problem, in particular, that the development of new antibiotics has slowed down in the 21st century [3]. The development and implantation of new and effective strategies to overcome the spread of antimicrobial resistance and decrease the resistance impact on public health and healthcare costs are needed [4,9].

The Antimicrobial stewardship (AMS) program is one of the most effective strategies to overcome bacterial resistance via taking various actions aimed at directly influencing antibiotic use, reducing unnecessary antibacterial prescriptions, enhancing clinical outcomes; and increasing safety [10,11,12]. As a result of antimicrobial resistance, the World Health Organization (WHO) developed the WHO Global Strategy for Containment of Antimicrobial Resistance [13]. That strategy aimed to control and reduce the spread of antimicrobial resistance and decrease the impact of resistance on public health and healthcare costs [13]. In 2015, the WHO announced a Global Action Plan (GAP) on antimicrobial resistance [13]. The GAP aimed to confirm the successful treatment and prevention of infectious diseases. The plan includes five strategic objectives [14].

In 2017, the WHO Model List of Essential Medicines developed the AWaRe classification of antibiotics: Access, Watch, and Reverse to improve accessibility and clinical outcomes while at the same time reducing antibiotic resistance and preserving the effectiveness of the Reserve (last resort) group. Access groups are a core set of antibiotics used as first- or second-line antibiotics, which are widely available and relatively safe. Antibiotics in the Watch group have higher toxicity and resistance compared with those in the Access group, and these antibiotics should be considered the primary focus of stewardship programs. The Reserve group is the last-resort choice used for specific indications when all alternative treatments have failed or are not proper [15,16]. The study aims to determine the effect of enhancing knowledge and awareness of the WHO Access, Watch, and Reserve (AWaRe) antibiotics classification on hospital clinical staff’s knowledge, attitudes and antibiotic prescribing practices.

## 2. Results

### 2.1. Demographic and Medical Characteristics of the Study Sample

In the pre-interventional stage, the questionnaire was filled out by 125 physicians and pharmacists. Of those, 18 questionnaires were excluded, bringing the included number to 107. More than half of the participants were males (*n* = 68, 63.6%), and 44.9% were <30 years old (Table 1). Most of the participants were specialists or consultant doctors, or resident doctors, who represented around 90% of the total sample. The participants tend to have different years of practice experience, with most of them (39.3%) having more than 8 years of experience in prescribing or dispensing medicines.

In the post-interventional stage, 117 questionnaires were completed and included. The participants had similar demographic profile as in the pre-interventional stage. More than half of the encounters were with male doctors and pharmacists (*n* = 75, 64.1%), and 45.3% were <30 years old. Most of the participants were specialists or consultant doctors (42.7%) and resident doctors (35.9%). The majority of the participants had more than eight years of practice in prescribing or dispensing medicines (41.9%; Table 1).

### 2.2. Knowledge, Perceptions, and Attitude about (AWaRe) Antibiotics Classification

All the participants responded to 14 statements regarding their knowledge, perceptions, and attitudes about (AWaRe) antibiotics classification. In the pre-interventional stage, more than three-quarters of the participants (78.5%) stated that they had not heard about the AWaRe classification of antibiotics before. After receiving the intervention, the percentage of those who heard about the AWaRe classification of antibiotics increased from 21.5% to 55.6% (Table 2).

**Figure 1:**
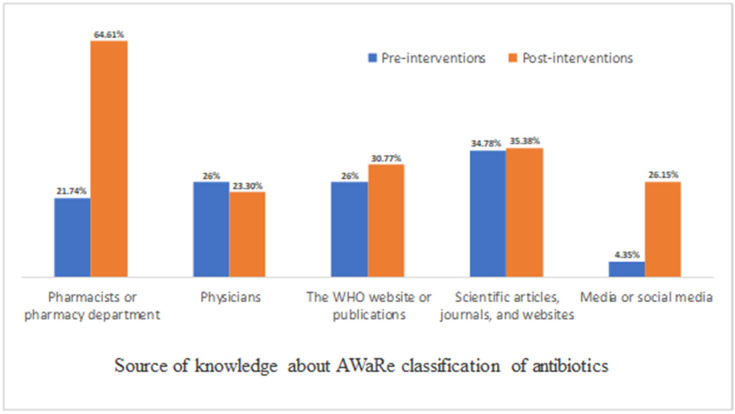
Main source of knowledge about AWaRe classification of antibiotics (they can choose more than one).

The sources of knowledge varied between pharmacists or pharmacy departments, physicians, scientific articles and journals, WHO publications and websites, and media and social media (Figure 1). In the pre-intervention phase, the most common sources of knowledge were scientific articles, journals, and websites (34.78%), physicians (26%), the WHO website or publications (26%), pharmacists or pharmacy department (21.74%), and media or social media (4.35%). In the post-intervention, the most common sources of knowledge were pharmacists or pharmacy departments (64.61%), scientific articles, journals, and websites (35.38%), the WHO website or publications (30.77%), physicians (20.3%), and media or social media (26.15%; Figure 1).

Assessment of attitude and perceptions about (AWaRe) antibiotics classification is provided in (Table 3). The percentage of participants who agree/strongly agree with the statement “I follow the AWaRe classification of antibiotics in my practice” increased from 21.74% to 58.46% after the intervention. Post intervention, the majority of respondents agree/strongly agree (58.46%) with the previous statement. However, before the intervention, the majority of respondents disagree/strongly disagree (39.14%) or were neutral (39.14%) regarding the previous statement.

Regarding the statement “The hospital’s regulations and guidelines encourage considering AWaRe classification of antibiotics in my practice”, the percentage of agree/strongly agree answers increased from 39.13% before the intervention to 70.77% after the intervention. The percentage of respondents who agree/strongly agree with the statement “I believe that following the AWaRe classification of antibiotics helps in reducing the rate of antibiotics resistance” increased from 82.16% to 87.69% after the intervention. Before the intervention, 56.52% of respondents thought that the AWaRe classification of antibiotics can suggest safe choices of antibiotics. This percentage increased to 90.77% after the intervention. In addition to that, 65.22% of participants before the intervention believed that AWaRe classification of antibiotics can suggest cost-effective choices of antibiotics. This percentage increased to 84.62% after the intervention. Finally, only 11.2% of respondents in the pre-intervention phase believed that training is needed on antibiotics resistance, antimicrobial stewardship, and AWaRe classification of antibiotics. In the post intervention, 95.38% of participants believed that training is needed on antibiotics resistance, antimicrobial stewardship, and AWaRe classification of antibiotics (Table 3).

### 2.3. Prescribed Antibiotics Measured by Global-PPS Method

In order to determine whether AWaRe antibiotic classification knowledge-enhancing intervention had influenced trends in prescribing antibiotics to inpatients, antibiotics’ DDDs/patients were calculated by using the Global-PPS method. Data on the amounts of prescribed antibiotics was collected from patients’ files in the ICU/CCU.

PPS data for a total of 18 inpatients in the pre-interventional stage was collected on Friday, 26 November 2021. Antibiotics were prescribed for 13 patients, all of them on that specific day. A second round of antibiotic PPS data collection was conducted on Friday, 18 March 2022. A total of 14 patients were in the ICU/CCU, and only 6 of them had been prescribed antibiotics (Table 4). The mean age of all ICU/CCU patients in the pre-interventional and post-interventional stages was 57.5 years and 52.9 years, respectively. More than 70% of them were male patients in both stages.

The rate of prescribing antibiotics in the pre-interventional stage was 4.36 DDDs/patients. This rate decreased to 2.12 DDDs/patient in the post-interventional stage. Around 70% of the prescribed DDDs of antibiotics in the pre-interventional stage belonged to the Watch category and 23.1% belonged to the Reserve category and only 7.6% belonged to the Access category. In the post-intervention stage, 49.5% of the prescribed DDDs of antibiotics were in the Watch category, 30.3% were in the Reserve category, and 20.2% were in the Access category.

The prescribed DDDs of antibiotics per patient decreased in the post-interventional stage from 3.02 to 1.05 for Watch antibiotics and from 1.01 to 0.64 for Reserve antibiotics. On the other hand, the prescribed DDDs of Access antibiotics per patient increased from 0.33 in the pre-intervention stage to 0.43 in the post-intervention stage.

### 2.4. Pharmacy Antibiotics Dispensing

A total of 40 antibiotics (or combinations) dispensed from the hospital pharmacy were categorized as the following: 9 in the Access group, 26 in the Watch group, and 5 in the Reserve group (Table 5). After the intervention, amoxicillin and co-amoxiclav (15.51%), and metronidazole (2.63%) had a higher proportion of the dispensing from the Access antibiotics group. Moreover, levofloxacin (14.89%), cefixime (14.31%), cefdinir (9.37%), ciprofloxacin (8.19%), and cefuroxime (7.66%) were the most dispensable antibiotics in the Watch group after the intervention. In addition to that, tigecycline (0.195%) was the most dispensed antibiotic from the Reserve group after the intervention.

Hospital antibiotic use of the Access group increased by 6.6% from pre- to post-intervention (from 22.53% to 24.01%). The use of the Watch group and Reserve group decreased post-intervention by 1.7% (from 77.05% to 75.73%), and 43.1% (from 0.42% to 0.25%), respectively (Table 6).

## 3. Discussion

This is a pre-post intervention study designed to assess the impact of improving the knowledge about WHO Access, Watch, and Reserve (AWaRe) antibiotics classification on healthcare practitioners’ knowledge, attitudes, and antibiotic prescribing practices. The provided educational interventions discussed the use of antibiotics that fall into each AWaRe category and the role of healthcare professionals in decreasing antibiotic resistance by using the AWaRe classification of antibiotics. The present study’s outcomes were comparable to the knowledge of healthcare providers before and after the intervention. Most participants in this study were males, with an average age of 30 years old. Furthermore, the majority of participants were specialists, consultant doctors, and resident doctors who had more than eight years of practice in prescribing or dispensing medicines in the 2 stages of the study.

Educational programs, training, and workshops provide healthcare practitioners with an opportunity to focus on the proper use of antibiotics and the complications of antibiotic resistance [17]. These educational programs could be in the form of posters and brochures, seminars and lectures, and media such as the internet and television [17]. A previous study conducted in Italy showed that medical students needed specific awareness of antibiotic use and resistance, plus specific training and courses about antibiotics in the core curriculum of the schools of medicine [18]. On 11 November 2021, the WHO submitted a survey; one of its issues was monitoring AMR activities by adapting the WHO’s “AWaRe” classification of antibiotics [19]. The data showed that 36% of 163 countries had adopted the AWaRe classification of antibiotics in their medicine list [19]. It also showed that the knowledge of the “AWaRe” tool increased compared to last year by 26% [19].

In this study, the intervention caused an increase in the percentage of clinical staff who have heard about the AWaRe antibiotic classification. Furthermore, the knowledge of the clinical staff regarding the meaning and purpose of AWaRe classification of antibiotics was improved after receiving the interventions. In addition to that, after the intervention, around 58% of respondents followed the AWaRe classification of antibiotics in their practice, while approximately 90.8% of respondents agreed that the AWaRe classification of antibiotics can suggest safe choices of antibiotics. It is clearly demonstrated from the aforementioned results that gaps in the educational background of healthcare practitioners regarding the AWaRe classification of antibiotics are present and need to be narrowed.

Antibiotic resistance is a global public health crisis in the 21st century that has become a health challenge for the WHO and antimicrobial stewardship programs [20]. The irrational and inappropriate use—including the overuse and misuse—of antibiotics increases the rate of resistance of bacteria and the emergence of antibiotic resistance [20]. The decline in the development of and research regarding newer antibiotics is critical in increasing attention [4]. At the same time, reserved usage and short-course antibiotics increased generic competition and cost. Furthermore, inevitable resistance affected the pharmaceutical companies’ profitability of antibiotic development [4].

According to a study in China, the sales of non-prescription antimicrobial drugs in low and middle-income countries reached up to 93% of total antimicrobial sales, with up to 100% of the sales of antibiotics without a prescription by pharmacists [21]. Many factors contribute to non-prescription sales of antibiotics, including pressure from the patient and the owner of the pharmacies, profit concerns, and patients’ socioeconomic status (many of them cannot afford physician fees) [21]. Similar findings were reported by a previous study that took place in Jordan to assess the extent of self-medication with antibiotics in a Jordanian population [22]. The data showed that Amoxicillin was the most commonly used antibiotic, and only 37.6% of patients followed the guidelines to take the correct dosage [22]. Moreover, 40.7% of Jordanian patients have taken antibiotics without a prescription, and 19.7% of patients have asked another doctor or physician to obtain antibiotics if the first doctor has not prescribed an antibiotic [22].

AMR has become a global health issue affecting developed and developing countries, including Jordan [18]. The misuse of antimicrobials is accelerating the resistance process due to the easy accessibility of antimicrobials and the lack of knowledge among physicians [18]. Therefore, antibiotic misuse and over-prescription consequences include a higher rate of antimicrobial resistance, higher cost, and increased risk of side effects [18]. A descriptive cross-sectional study was undertaken in the second-largest population province of China (2012–2019) to analyze the changes in patterns of antibiotic consumption by WHO AWaRe classification in health care institutions in high-income areas (HIAs) and the upper-middle-income areas (UMIAs) [23]. The data showed that the antibiotic consumption rate was lower in the HIAs than in the UMIAs [23]. The consumption of antibiotics in the access group continuously decreased from 50% to 44.9%, whereas the consumption in the watch group increased from 42 to 45.2% [23]. At the same time, the consumption rate of linezolid and tigecycline, which belong to the Reserve group, increased in Shandong. In this study, the percentage of antibiotic consumption for the Access group increased by 6.6%. On the other hand, the percentage of antibiotic consumption for the Watch and Reserve groups decreased by 1.7% and 43.1% after the intervention.

In one study, Chae and colleagues evaluated the impact of the implementation of the National Action Plan (NAP) on antibiotic consumption in South Korea [24]. The NAP consisted of numerous educational campaigns targeted toward enhancing knowledge regarding AMR and the AWaRe antibiotic classification in addition to other measures like enhancing cooperation and coordination between sectors [24]. A decrease in the total consumption of antibiotics belonging to the Access and Watch group was observed [24]. In a study designed to measure the effect of antibiotic control and education programs on antibiotic consumption, Apisarnthanarak and colleagues compared the rate of antibiotic use a year before and a year after the intervention [25]. They found that the rate of antibiotic consumption significantly decreased by 24% (*p* < 0.001) after the intervention [25]. According to a study conducted in Punjab, Pakistan to evaluate antibiotic consumption among neonates and children, the most common indications for antibiotic use were respiratory tract infections, sepsis, and prophylaxis for medical problems [26]. The consumption of antibiotics in the Access categories was 49.5%, and the most commonly prescribed antibiotics were ceftriaxone (24.2%), ampicillin (16.7%), and amikacin (23.2%) [26]. The consumption in the Watch categories was 45.5% and no antibiotics were prescribed from the ‘Reserved’ category [26].

In the present study, amoxicillin and co-amoxiclav (15.51%), and metronidazole (2.63%) had the highest proportions of dispensing from the Access antibiotics group. Moreover, levofloxacin (14.89%), cefixime (14.31%), cefdinir (9.37%), ciprofloxacin (8.19%), and cefuroxime (7.66%) were the most dispensable antibiotics in the Watch group. In addition to that, tigecycline (0.195%) was the most dispensed antibiotic from the Reserve group. Similar findings were observed in a study that was conducted to evaluate community-level antibiotic consumption in a rural area in Vietnam according to the WHO AWaRe groups [27]. The aforementioned study showed that the most frequently consumed antibiotics in the Access categories were amoxicillin, ampicillin, and cefalexin (first-generation cephalosporins), which accounted for around 60% of total antibiotic consumption [27]. Furthermore, the consumption in the watch group of antibiotics was about 40% of second-and third-generation cephalosporins (cefuroxime, cefdinir, cefixime, and cefpodoxime), fluoroquinolones (levofloxacin and ciprofloxacin), and macrolides (azithromycin, clarithromycin, and erythromycin) [27].

This study was strengthened by the fact it is the first one to evaluate the impact of AWaRe tool-targeted educational programs on the total consumption of antibiotics. However, the study has some limitations. Firstly, the study was conducted in a specific healthcare setting, i.e., a private hospital and only one hospital. The study would benefit from being repeated in other hospitals (e.g., public and teaching hospitals) to improve the generalizability of the findings. In addition, the follow-up period post-intervention did not allow for capturing the sustainability of the intervention. Further research with longer follow-up study periods is needed.

In conclusion, the study aims to determine the effect of enhancing knowledge and awareness of the WHO Access, Watch, and Reserve (AWaRe) antibiotics classification on hospital clinical staff’s knowledge, attitudes and antibiotic prescribing practices. The designed educational intervention increased the knowledge of participants regarding AWaRe antibiotic classification. Furthermore, the percentage of participants who follow the AWaRe classification of antibiotics was increased. The total consumption of antibiotics was increased for antibiotics in the Access group and was decreased for antibiotics in the Watch and Reserve groups. This study revealed improvement in healthcare practitioners’ perception regarding the AWaRe classification of antibiotics can suggest safe choices of antibiotics. This study showed important gaps in knowledge and attitudes towards AWaRe, highlighting the need for increasing the awareness of the AWaRe tool amongst healthcare practitioners to ensure rational use of antibiotics.

## 4. Methods

### 4.1. Study Design and Sample Size

This is a pre-post intervention study that was conducted between September 2021 and March 2022 to evaluate the effect of knowledge and awareness about the AWaRe classification on prescribing antibiotics in The Specialty Hospital in Amman, Jordan. The Specialty Hospital is accredited by the Joint Commission International (JCI) and the Health Care Accreditation Council (HCAC) with a 265-bed capacity and different medical departments. According to the interventional education regarding AWaRE classification, the study was divided into two phases, i.e., pre-interventional (initial phase) and post-interventional (second phase).

The intervention was in the form of an educational lecture designed to introduce, teach, or remind the physicians, clinical pharmacists, and pharmacists about the AWaRe classification of antibiotics and the risk of antibiotic resistance. It also included a presentation of the antimicrobial prescribing guidelines in the hospital.

### 4.2. Pre-Intervention Phase

In September 2021, a questionnaire was distributed by Google form and paper form to assess the knowledge of physicians, clinical pharmacists, and pharmacists about the AWaRe classification of antibiotics (Supplementary Materials (Appendix S1)). The questionnaires were collected from 10 September to 10 November 2021.

Simultaneously, data from the pharmacy access system were collected for antibiotic orders for inpatients and emergency department patients from the 1 September until 30 November 2021, and a point prevalence survey (PPS) [28,29] was performed on 26 November 2021 for inpatients in ICU/CCU departments (Figure 1).

### 4.3. Educational Interventions

In December 2021, the educational intervention (presentation) was done using the Zoom application. In addition, the content of the latter educational presentation was used for face-to-face discussion intervention. The virtual and face-to-face interventions targeted the hospital’s physicians, clinical pharmacists, and pharmacists’ knowledge about the AWaRe classification of antibiotics. The delivered interventions included several aspects related to antibiotic use and antibiotics resistance, such as the definition and factors influencing the development of antimicrobial resistance (main driver is the misuse and overuse of antibiotics in humans); misuse and inappropriate use of antibiotics (e.g., unnecessary antibiotic consumption, the overuse of broad spectrum antibiotics); the WHO global action plan and strategy for containment of antimicrobial resistance; the WHO Model List of Essential Medicines and the WHO AWaRe classification of antibiotics; description of each of the WHO AWaRe antibiotic group (Access, Watch, Reserve), examples of included antibiotic agents and the uses of antibiotics that fall into each category; the role of physicians, and pharmacists in decreasing antibiotic resistance by using the AWaRe classification of antibiotics. The role of physicians involved prescribing the antibiotics according to the guidelines, switching to a narrow spectrum where appropriate, educating patients about antimicrobial resistance, and following appropriate antibiotic prescribing practices. The role of pharmacists involved dispensing antibiotics in a safe and rational manner, spreading awareness about the dangers of inappropriate and misuse use of antibiotics, and not dispensing antibiotics without a prescription. The role of institutions and health organizations, especially the role of the Jordanian Ministry of Health (JFDA), in reducing unnecessary consumption of antibiotics was also discussed.

### 4.4. Post-Intervention Phase

**Figure 2:**
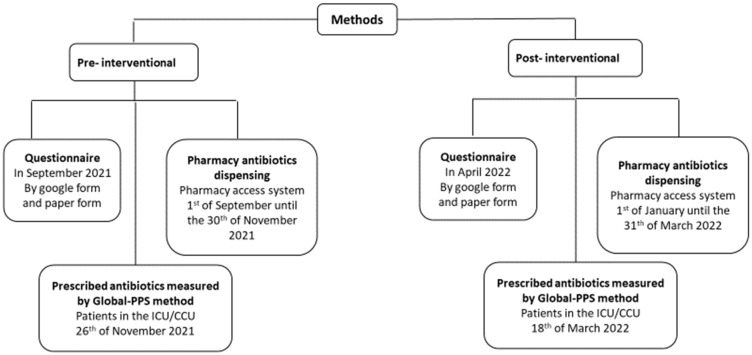
Schematic presentation showing the steps of data collection.

After providing the educational intervention, the data from the pharmacy access system was re-collected from 1 January until 31 March 2021 for antibiotic orders for inpatients, and Global-PSS was conducted on the 18 March 2022 in the same ICU/CCU departments (Figure 2).

In April 2022, a questionnaire was distributed by Google form and paper form to re-evaluate the knowledge of the physicians, clinical pharmacists, and pharmacists about the AWaRe classification of antibiotics after the interventions (Supplementary Materials (Appendix S1)). The questionnaires were filled out and collected over the period between 10 April and 15 May 2022.

### 4.5. Data Collection

Figure 2 represents the three sets of data that were collected in over the two phases of the study. The phases were pre-interventional (initial phase) and post-interventional. The types of data are described as the following:

Firstly, the knowledge about the AWaRE classification of antibiotics assessing questionnaire, which was divided into: (1) Demographic and medical characteristics of respondents, that involved information about the hospital’s physicians, clinical pharmacists, and pharmacists’ gender, age, profession, and years of practice in prescribing or dispensing medicines. (2) Knowledge, perceptions, and attitudes toward the classification of AWaRe antibiotics, with responses as (1: strongly agree, 2: agree, 3: neutral, 4: disagree, and 5: strongly disagree).

Secondly, antibiotics-dispensing data for all admissions. This data was retrieved from the pharmacy inventory computer system (pharmacy access) for three months retrospectively and three months post-intervention, The type of data was quantities of antibiotic units that were dispensed to different hospital departments, i.e., the internal medicine, orthopedics, ICU/CCU, and emergency departments. The data covered a 3-month period before the intervention and the same duration after the intervention. 

Thirdly, antibiotic prescribing data for inpatients in the Intensive Care Unit (ICU), Coronary Care Unit (CCU), Intermediate Care Unit, and Cardiac care unit was collected according to the Global-PPS procedure.

### 4.6. Defined Daily Dose (DDD)

The DDD is the assumed average maintenance dose per day for a drug used for its main indication in adults [30]. The DDD is a unit of measurement and does not necessarily reflect the recommended or prescribed. Antibiotics dispensing data was calculated as a DDD per 100 patient days. Results were analyzed and presented as per AWaRe classification.

### 4.7. Global-PPS

A pre-prepared standard data collection (www.global-pps.com; accessed on 1 November 2021) form for each enrolled patient was used to fill in the necessary data: (i) ward form: the data was collected from the patient’s medical file, including date of survey, auditor code, hospital name, ward name, ward type, and mixed ward; (ii) patient form: the demographic data was collected from the patient’s medical file including the patient identifier, age, gender, insurance status.; and (iii) antimicrobial level data.

### 4.8. Statistical Analysis

The collected data were coded, entered, and analyzed using the Statistical Package for Social Sciences (SPSS) version 22. The descriptive analysis was conducted using mean and standard deviation (SD) for continuous variables and frequency (percentages) for categorical variables. Antibiotics were classified as per the WHO AWaRe classification (Access, Watch, and Reserve) of antimicrobial usage [31]. Relative rate of change is calculated as DDD per 100 patient days% in the post-intervention/DDD per 100 patient days% in the pre-intervention.

## Figures and Tables

**Table 1:** Demographic and medical characteristics of participants included in the study.

	Pre-Interventional	Post-Interventional
Parameter	*n* (%)	*n* (%)
**Age (years)**		
○ <30	48 (44.9)	53 (45.3)
○ 30–39	19 (17.8)	19 (16.2)
○ 40–49	16 (15.0)	17 (14.5)
○ ≥50	24 (22.4)	28 (23.9)
**Gender**		
○ Male	68 (63.6)	75 (64.1)
○ Female	39 (36.4)	42 (35.9)
**Profession**		
○ Specialist or consultant doctors	48 (44.9)	50 (42.7)
○ Resident doctors	49 (45.8)	42 (35.9)
○ Clinical pharmacists	0 (0)	5 (4.3)
○ Pharmacists	10 (9.3)	20 (17.1)
**Years of practice in prescribing or dispensing medicines**		
○ 1–2 years	32 (29.9)	29 (24.8)
○ 3–4 years	13 (12.1)	22 (18.8)
○ 5–7 years	20 (18.7)	17 (14.5)
○ More than 8 years	42 (39.3)	49 (41.9)

**Table 2:** Assessment of knowledge about (AWaRe) antibiotics classification.

	Pre-Interventional	Post-Interventional
Parameter	*n* (%)	*n* (%)
**Have you heard about (AWaRe) Classification of antibiotics?**		
○ Yes	23 (21.5)	65 (55.6)
○ No	84 (78.5)	52 (44.4)
**I have a good knowledge regarding the meaning and purpose of AWaRe classification of antibiotics**		
○ Agree/Strongly agree	9 (39.13)	49 (75.38)
○ Disagree/Strongly disagree	5 (21.74)	5 (7.69)
○ Neutral	9 (39.13)	11 (16.92)

**Table 3:** Assessment of attitude and perceptions about (AWaRe) antibiotics classification.

	Pre-Interventional	Post-Interventional
Parameter	*n* (%)	*n* (%)
**I follow the AWaRe classification of antibiotics in my practice.**		
○ Agree/Strongly agree	5 (21.74)	38 (58.46)
○ Disagree/Strongly disagree	9 (39.14)	6 (9.23)
○ Neutral	9 (39.14)	21 (32.31)
**The hospital’s regulations and guidelines encourage considering AWaRe classification of antibiotics in my practice**		
○ Agree/Strongly agree	9 (39.13)	46 (70.77)
○ Disagree/Strongly disagree	8 (34.78)	2 (3.08)
○ Neutral	6 (26.09)	17 (26.15)
**I believe that following the AWaRe classification of antibiotics helps in reduction the rate of antibiotics resistance**		
○ Agree/Strongly agree	19 (82.16)	57 (87.69)
○ Disagree/Strongly disagree	2 (8.70)	4 (6.15)
○ Neutral	2 (8.70)	4 (6.15)
**The AWaRe classification of antibiotics is compatible with the scientific knowledge I have gain**		
○ Agree/Strongly agree	16 (69.57)	56 (86.15)
○ Disagree/Strongly disagree	2 (8.70)	3 (4.62)
○ Neutral	5 (21.74)	6 (9.23)
**More insight should be excreted on promoting AWaRe classification of antibiotics**		
○ Agree/Strongly agree	19 (82.61)	56 (86.15)
○ Disagree/Strongly disagree	2 (8.7)	3 (4.62)
○ Neutral	2 (8.7)	6 (9.23)
**I believe that AWaRe classification of antibiotics can suggest safe choices of antibiotics**		
○ Agree/Strongly agree	13 (56.52)	59 (90.77)
○ Disagree/Strongly disagree	2 (8.7)	2 (3.08)
○ Neutral	8 (34.78)	4 (6.15)
**I believe that AWaRe classification of antibiotics can suggest cost- effective choices of antibiotics**		
○ Agree/Strongly agree	15 (65.22)	55 (84.62)
○ Disagree/Strongly disagree	1 (4.34)	3 (4.62)
○ Neutral	7 (30.43)	7 (10.77)
**Training is needed on antibiotics resistance, antimicrobial stewardship, and AWaRe classification of antibiotics**		
○ Agree/Strongly agree	12 (11.2)	62 (95.38)
○ Disagree/Strongly disagree	4 (3.7)	1 (1.54)
○ Neutral	7 (6.5)	2 (3.08)

**Table 4:** Characteristics of patients and rate of antibiotic dispensation in the ICU/CCU in Global-PSS.

	Pre-Interventional	Post-Interventional
Parameter	(*n* = 18)	(*n* = 14)
**Mean age** (SD)	57.5 (±6.3)	52.9 (±6.5)
**Gender**		
○ Male	14 (77.8%)	10 (71.4%)
○ Female	4 (22.2%)	4 (28.6%)
**Prescribed Access antibiotics** (DDDs/patient)	0.33 (7.6%)	0.43 (20.2%)
**Prescribed Watch antibiotics** (DDDs/patient)	3.02 (69.3%)	1.05 (49.5%)
**Prescribed Reserve antibiotics** (DDDs/patient)	1.01 (23.1%)	0.64 (30.3%)
**Prescribed total antibiotics** (DDDs/patient)	4.36	2.12

**Table 5:** DDDs per 100 patient days of antibiotics dispensed by the hospital pharmacy.

	Pre-Interventional		Post-Interventional	
	DDDs/100 Patient Days	% of Total Antibiotic Use	DDDs/100 Patient Days	% of Total Antibiotic Use
**Access**				
Amikacin	1.326	0.684	1.219	0.569
Amoxicillin and Co-amoxiclav	27.357	14.105	33.215	15.507
Ampicillin	0.290	0.150	0.333	0.155
Cefalexin	0.116	0.060	0.222	0.104
Cefazolin	2.428	1.252	3.362	1.570
Clindamycin	2.932	1.512	3.003	1.402
Doxycycline	0.853	0.440	3.736	1.744
Gentamicin	0.753	0.388	0.712	0.332
Metronidazole	7.633	3.936	5.636	2.631
**Watch**				
Azithromycin	5.693	2.935	7.320	3.417
Cefaclor	1.314	0.677	2.320	1.083
Cefdinir	21.858	11.270	20.061	9.366
Cefditoren	1.629	0.840	1.245	0.581
Cefixime	25.022	12.901	30.664	14.316
Cefotaxime	0.878	0.453	0.818	0.382
Cefpodoxime	5.013	2.585	3.269	1.526
Cefprozil	1.745	0.900	1.290	0.602
Ceftazidime	0.950	0.490	0.845	0.394
Ceftizoxime	4.064	2.095	3.965	1.851
Ceftriaxone	5.893	3.038	6.117	2.856
Cefuroxime	15.749	8.120	16.417	7.664
Ciprofloxacin	19.703	10.159	17.545	8.191
Clarithromycin	1.249	0.644	1.548	0.723
Erythromycin	0.322	0.166	0.365	0.170
Ertapenem	1.226	0.632	1.245	0.581
Imipenem/Cilastatin	0.812	0.419	0.938	0.438
Levofloxacin	24.501	12.633	31.902	14.894
Lincomycin	0.092	0.047	0.097	0.045
Meropenem	1.834	0.946	2.855	1.333
Moxifloxacin	4.997	2.576	6.288	2.936
Piperacillin/Tazobactam	1.290	0.665	1.344	0.627
Teicoplanin	1.881	0.970	1.810	0.845
Vancomycin	1.687	0.870	1.883	0.879
Cefepime	0.043	0.022	0.018	0.008
**Reserve**				
Ceftazidime/Avibactam	0.084	0.043	0.044	0.021
Ceftobiprole	0.013	0.007	0.006	0.003
Colistimethate	0.121	0.062	0.050	0.023
Linezolid	0.019	0.010	0.0	0.000
Tigecycline	0.582	0.300	0.418	0.195
**Total antibiotics**	**193.96**	**100**	**214.20**	**100**

**Table 6:** Percentage of total antibiotic consumption (DDDs) by WHO AWaRe category (Access/Watch/Reserve) for pre/post intervention.

	Access	Watch	Reserve
	DDD/100 patient days (%)		
Pre-intervention	43.69 (22.53)	149.45 (77.05)	0.82 (0.42)
Post-intervention	51.44 (24.01)	162.22 (75.73)	0.54 (0.25)
Relative rate of change *	1.066	0.983	0.569

* Relative rate of change is calculated as DDD per 100 patient days% in the post-intervention/DDD per 100 patient days% in the pre-intervention.

## Data Availability

The data is contained in the article.

## Supplementary Materials

The following supporting information can be downloaded at: https://www.mdpi.com/article/10.3390/antibiotics12060951/s1, Appendix S1. Survey on Knowledge and Perceptions about (AWaRe) Antibiotics Classification.
