# Supplementary material for: An Evaluation of the Impact of Increasing the Awareness of the WHO Access, Watch, and Reserve (AWaRe) Antibiotics Classification on Knowledge, Attitudes, and Hospital Antibiotic Prescribing Practices
Source: Antibiotics (Basel). 2023 May 23;12(6):951. doi: 10.3390/antibiotics12060951 (PMC10295604; doi:10.3390/antibiotics12060951)
Supplement: Supplementary file 1 [file antibiotics-12-00951-s001.zip › antibiotics-2357024-supplementary.pdf]

Appendix 1. Survey on Knowledge and Perceptions about (AWaRe) Antibiotics Classification

Determining the Effect of Knowledge of WHO Access, Watch, and Reserve (AWaRe) Antibiotics Classification on their Hospital Prescribing

Dear participant

A researcher from the Applied Science Private University is carrying out a research project to determine the Effect of Knowledge of WHO Access, Watch, and Reserve (AWaRe) Antibiotics Classification on their Hospital Prescribing.

Your participation in this research study is voluntary, and you may choose not to participate. If you decide to participate in this research survey, you may withdraw at any time.

We would like to confirm that all information provided here will be kept confidential. All data will be stored in a password-protected electronic format. To help protect your confidentiality, the surveys will not contain information that will personally identify you, and will be used only for research purposes.

The procedure involves filling an online survey that will take approximately 10 minutes. Your participation in completing this survey is highly appreciated.

ELECTRONIC CONSENT: Please select your choice below.

Clicking on the "agree" button below indicates that:

1.You have read the above information

2.You voluntarily agree to participate

If you do not wish to participate in the research study, please decline participation by clicking on the "disagree" button.

☐Agree

☐Disagree

Part 1: Demographics information

1. **Age (Years)**:
   - ☐ <30
   - ☐ 30 – 39

- 40 – 49
- ≥ 50

2. **Gender:**
   - Male
   - Female
3. **Profession:**
   - Specialist or consultant doctor
   - Resident doctor
   - Clinical pharmacists
   - Pharmacists
4. **Years of practice in prescribing or dispensing medicines:**
   - 1- 2 years
   - 3 - 4 years
   - 5 - 7 years
   - More than 8 years

Part 2: Knowledge and perceptions about (AWaRe) Antibiotics Classification

* If your answer is No, please submit the form.

* If your answer is Yes, please continue to the next question.

1. **Have you heard about (AWaRe) Classification of antibiotics?**
   - o Yes
   - o No
2. **If your answer to the previous question was yes, please choose the source you heard about them from. (You can select more than one choice)**
   - o Pharmacists or pharmacy department
   - o Physicians
   - o The WHO website or publications
   - o Scientific articles, journals, and websites
   - o Media or social media
3. **I have a good knowledge regarding the meaning and purpose of AWaRe classification of antibiotics.**

- Strongly agree
- Agree
- Neutral
- Disagree
- Strongly disagree

4. **I follow the AWaRe classification of antibiotics in my practice.**
   - Strongly agree
   - Agree
   - Neutral
   - Disagree
   - Strongly disagree
5. **The hospital's regulations and guidelines encourage considering AWaRe classification of antibiotics in my practice.**
   - Strongly agree
   - Agree
   - Neutral
   - Disagree
   - Strongly disagree
6. **I believe that following the AWaRe classification of antibiotics helps in reduction the rate of antibiotics resistance.**
   - Strongly agree
   - Agree
   - Neutral
   - Disagree
   - Strongly disagree
7. **The AWaRe classification of antibiotics is compatible with the scientific knowledge I have gain.**
   - Strongly agree
   - Agree
   - Neutral
   - Disagree
   - Strongly disagree

8. More insight should be excreted on promoting AWaRe classification of antibiotics.

- Strongly agree
- Agree
- Neutral
- Disagree
- Strongly disagree

9. I believe that AWaRe classification of antibiotics can suggest safe choices of antibiotics.

- Strongly agree
- Agree
- Neutral
- Disagree
- Strongly disagree

10. I believe that AWaRe classification of antibiotics can suggest cost-effective choices of antibiotics.

- Strongly agree
- Agree
- Neutral
- Disagree
- Strongly disagree

11. Training is needed on antibiotics resistance, antimicrobial stewardship, and AWaRe classification of antibiotics.

- Strongly agree
- Agree
- Neutral
- Disagree
- Strongly disagree
